# Susceptibility Patterns in Clinical Isolates of *Mycobacterium avium* Complex from a Hospital in Southern Spain

**DOI:** 10.3390/microorganisms12122613

**Published:** 2024-12-17

**Authors:** Adrián González Martínez, María Aguilera, María Tarriño, Ana Alberola, Juan Antonio Reguera, Antonio Sampedro, Jose María Navarro, Javier Rodríguez Granger

**Affiliations:** Servicio de Microbiología, University Hospital Virgen de las Nieves, 18014 Granada, Spain; maria.aguilera.franco.sspa@juntadeandalucia.es (M.A.); mariatarrinoleon@gmail.com (M.T.); anaalberolaromano@gmail.com (A.A.); jantonio.reguera.sspa@juntadeandalucia.es (J.A.R.); antonioj.sampedro.sspa@juntadeandalucia.es (A.S.); josem.navarro.sspa@juntadeandalucia.es (J.M.N.); javierm.rodriguez.sspa@juntadeandalucia.es (J.R.G.)

**Keywords:** MAC, susceptibility profiles, antibiotics, *Mycobacterium*, AST

## Abstract

The incidence of infections caused by the *Mycobacterium avium* complex (MAC) has risen significantly, posing diagnostic and therapeutic challenges. This study analyzed 134 clinical isolates of the *Mycobacterium avium* complex from southern Spain, performing in vitro antimicrobial susceptibility testing using a commercial microdilution technique to generate additional data, refine treatment strategies, and improve patient outcomes. Phenotypic susceptibility testing revealed clarithromycin and amikacin as the most effective antibiotics, with susceptibility rates exceeding 90%, while linezolid and moxifloxacin exhibited limited activity, with resistance rates of 49.3% and 41.8%. A comparative analysis between *M. avium* and *M. intracellulare* showed significant differences in resistance to amikacin and linezolid, with *M. avium* exhibiting higher resistance rates. Additionally, species-specific differences were observed in MIC distributions for ethionamide, ciprofloxacin, and streptomycin. Our data reveal regional variability in resistance patterns, particularly for moxifloxacin and linezolid, which exhibit differing resistance rates compared to studies from other regions. The significant MIC differences for several antibiotics between *M. avium* and *M. intracellulare* underscore the importance of species-level identification and the heterogeneity in resistance mechanisms within MAC.

## 1. Introduction

In recent years, the incidence and clinical significance of non-tuberculous mycobacteria (NTM) infections have noticeably increased, especially among patients with chronic pulmonary disease [1]. There are over 200 different species of NTM, with *Mycobacterium avium* complex (MAC) species being the most prevalent [2,3]. MAC comprises several species and subspecies, with *M. avium*, *M. intracellulare*, and *M. chimaera* being the most significant [4]. The *M. avium* species has been classified into four distinct subspecies: *M. avium* subsp. *avium*, *M. avium* subsp. *hominissuis*, *M. avium* subsp. *paratuberculosis*, and *M. avium* subsp. *silvaticum* [5]. The most clinically relevant subspecies in humans is *M. avium* subsp. *hominissuis*, while the other subspecies are primarily associated with infections in animals [6]. These organisms are commonly found in water, house dust, and soil. Once they reach plumbing systems in homes and buildings, their hydrophobic nature enables them to adhere to surfaces. While patient-to-patient transmission of pulmonary MAC is rare, infection clusters often originate from a shared water source, suggesting that the inhalation of infectious aerosols is the primary mode of transmission leading to pulmonary disease. Additionally, MAC can enter the body through the gastrointestinal tract or via direct inoculation resulting from trauma or invasive procedures [7].

The most significant clinical manifestation of MAC infection is pulmonary disease, which may present in three forms: fibrocavitary, nodular/bronchiectatic, and hypersensitivity pneumonitis (hot tub lung). Other clinical manifestations caused by MAC include the invasion of the lymph nodes, bones, joints, skin, and soft tissue, as well as the potential for systemic dissemination [7,8]. Pulmonary disease primarily occurs in immunocompetent patients with underlying chronic pulmonary conditions; lymphadenitis predominantly affects children, and disseminated disease is most commonly observed in patients with advanced-stage AIDS or lymphoma. Managing MAC infections poses a significant challenge in clinical practice due to the complexities involved in their diagnosis and treatment. According to the Infectious Diseases Society of America (IDSA) criteria, diagnosing MAC pulmonary disease requires not only clinical and radiological findings but also the isolation of the microorganism in two or more sputum samples or a single sterile specimen, as a single positive sputum sample may indicate environmental contamination or transient colonization [9].

Once the diagnosis is established, treatment is challenging. Antibiotic therapy is not always necessary, but when it is, it requires the use of multiple antimicrobial agents. These agents are often associated with clinically significant adverse reactions and must be administered for prolonged periods [9]. The cornerstone of treatment is macrolides, which are recommended to be used in combination with rifampicin and ethambutol, with or without a fourth agent, such as amikacin [10,11]. Other agents that may exhibit activity include moxifloxacin and linezolid, but their clinical efficacy for MAC has not been established. Antimicrobial susceptibility testing (AST) is recommended for clinically significant isolates, but the main drawback that can be found is that there is a clinical correlation with in vitro MICs only for macrolides and, most recently, amikacin [11]. International guidelines for the treatment of MAC infections do not differentiate treatment at the species level [9]. However, previous studies have demonstrated significant differences in resistance patterns among MAC species [12,13].

In the southern region of Spain, data on the local susceptibility patterns of individual MAC species or MAC in general can scarcely be found. To the best of our knowledge, no studies specifically addressing this topic have been conducted in this region. Therefore, we collected clinical isolates of MAC and conducted in vitro antimicrobial susceptibility testing (AST) to generate additional data and contribute to improving treatment strategies and patient outcomes.

## 2. Materials and Methods

### 2.1. Sample Collection

One hundred and thirty-four strains of MAC were collected from various clinical samples between 2019 and 2024 at the Microbiology Department of Virgen de las Nieves Hospital (Granada, Spain). The Virgen de las Nieves Hospital serves as the reference center for mycobacteria studies in the province. Consequently, samples from all hospitals and primary care centers across the region are received. The clinical specimens included respiratory samples, such as sputum and bronchoalveolar lavage, as well as extrarespiratory samples, regardless of whether the isolation was associated with disease. Only one isolate per patient was included in the study.

### 2.2. Microbiological Methods

Mycobacterial cultures were performed using automated liquid media systems: MGIT, BACTEC 960 system (Becton Dickinson, Franklin Lakes, NJ, USA), and VersaTREK culture system (Thermo Fisher Scientific, Waltham, MA, USA). Complex identification was performed on liquid media using the commercial kit GenoType Mycobacterium CM (Hain LifeScience, Nehren, Germany) and species-level identification was conducted using the kit GenoType NTM-DR (Hain Lifescience). This kit was also used to detect genotypic resistance to macrolides and aminoglycosides by identifying mutations in the *rrl* (23S rRNA) and *rrs* (16S rRNA) genes, respectively. Subsequently, subculturing was performed on solid media (NTM elite agar and blood agar, bioMérieux, Marcy-l’Étoile, France) before conducting AST. Additionally, species identification was confirmed through MALDI-TOF mass spectrometry (Bruker, Bremen, Germany) on solid media.

AST was performed using the commercial microdilution technique with Sensititre Myco SLOMYCOI AST plates (Thermo Fisher Scientific) according to the manufacturer’s instructions. The antibiotics included in the kit are clarithromycin, linezolid, moxifloxacin, amikacin, rifabutin, ethambutol, isoniazid, rifampicin, trimethoprim-sulfamethoxazole (SXT), ciprofloxacin, streptomycin, doxycycline, and ethionamide. The interpretation of clarithromycin, moxifloxacin, linezolid, and amikacin susceptibility was based on the breakpoints provided by the CLSI Guidelines [14]. The remaining antibiotics lack CLSI-defined breakpoints; therefore, the analysis was conducted using MIC distributions. As a reference strain for quality control, *M. avium* ATCC 25291 was used.

### 2.3. Statistical Analysis

Statistical analysis was performed using RStudio version 4.3.3. The frequency and percentage of isolates for each MAC species were calculated, along with the distribution of clinical samples (respiratory vs. extrarespiratory). The number and percentage of isolates resistant and susceptible to each antibiotic were summarized. Chi-square or Fisher’s exact tests were used to compare susceptibility profiles between species. Statistically significant differences were considered for *p* < 0.05. For the remaining antibiotics, we calculated the median MIC values within each species group. To compare the MIC distributions between the two species, the Wilcoxon rank-sum test (also known as the Mann–Whitney U test) was applied, as the Shapiro–Wilk test indicated non-normal distributions (*p* < 0.0001 for all antibiotics). *p*-values from the Wilcoxon test were reported for each antibiotic to determine if significant differences in MIC values existed between the two species. A threshold of *p* < 0.05 was considered to be statistically significant.

## 3. Results

Phenotypic antimicrobial susceptibility testing (AST) was performed on 134 MAC strains, including 66 *M. avium* and 68 *M. intracellulare*. Of the 134 MAC isolates, 128 (92.8%) were derived from respiratory samples, with the majority being sputum samples (104 isolates, 81.3%) and the remainder obtained via bronchoscopy. Only six isolates were from extrapulmonary samples, comprising four lymph node biopsies and two blood cultures.

Regarding demographic data, 55% of the patients were female, and 45% were male, with a median age of 63 years.

The antibiotics with breakpoints provided by the CLSI Guidelines are clarithromycin, linezolid, moxifloxacin, and amikacin. The distribution of isolates, expressed as the percentage of susceptibility, intermediate, or resistance to each antibiotic and each species, is presented in Table 1.

The antibiotic exhibiting the highest sensitivity percentage was clarithromycin, with a sensitivity rate of 98.5%, followed by amikacin, which demonstrated a sensitivity rate of 91%. Of the two *M. avium* isolates resistant to clarithromycin, both exhibited mutations in the *rrl* gene. In contrast, among the strains classified as resistant or intermediate to amikacin, only three showed mutations in the *rrs* gene.

Linezolid and moxifloxacin exhibited much lower sensitivity percentages, with rates of 10.4% and 20.9%, respectively. However, approximately one-third of the isolates were categorized as intermediate, with rates of 40.3% and 37.3%, respectively.

**Figure 1:**
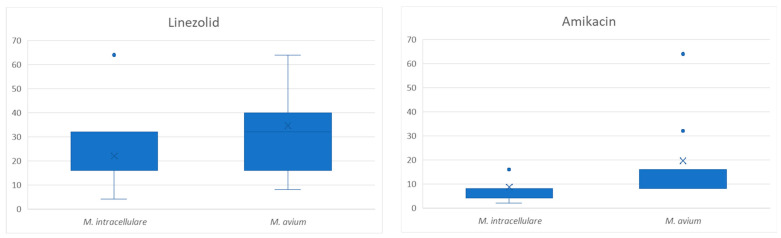
Boxplots graphs showing the MIC distribution of linezolid and amikacin for *M. intracellulare* and *M. avium*. Boxplots represent the interquartile range (box) and values within 1.5× IQR (whiskers). Outliers are shown as individual points, and the mean is represented by an ‘X’.

Regarding inter-species differences, no significant differences were found for clarithromycin and moxifloxacin. However, significant differences were observed for amikacin [*p* = 0.000001] and linezolid [*p* = 0.001], with *M. avium* showing a significantly higher resistance to both antibiotics. In Figure 1, boxplot graphs are shown to compare the MIC (minimum inhibitory concentrations) distributions for both antibiotics.

The median MIC values for the remaining antibiotics, stratified by species, are presented in Table 2, along with *p*-values from the Wilcoxon rank-sum test to assess differences between species.

Most antibiotics exhibited similar MICs across both species. However, ethionamide, ciprofloxacin, and streptomycin showed statistically significant differences between them. Specifically, the MIC for streptomycin and ciprofloxacin was higher in *M. avium*, while the MIC for ethionamide was higher in *M. intracellulare*.

## 4. Discussion

As previously mentioned, infections caused by non-tuberculous mycobacteria (NTM), particularly those by the *Mycobacterium avium* complex (MAC) have significantly increased in recent years [1,2,3]. These infections often present challenges in diagnosis and, especially, in treatment [9]. Due to the treatment challenges, the CLSI recommends performing antimicrobial susceptibility testing (AST) for all patients with MAC-pulmonary disease prior to initiating treatment, although the in vitro–in vivo correlation has only been established for a few antibiotics [11,14]. To our knowledge, few studies in our region have performed susceptibility testing for MAC to assess the epidemiology of resistance. In other regions, there are several studies available, but resistance profiles may vary by region, making it essential to provide further data to enhance treatment approaches. This study provides valuable data on the antimicrobial susceptibility profiles of MAC strains isolated in southern Spain, thereby contributing to the growing body of literature.

The main study conducted in Spain was realized by Fernandez-Pittol et al., which compares the susceptibility patterns of the three main species of the MAC complex in isolates from three hospitals in Barcelona (northeastern Spain) [12]. Among the antibiotics with established CLSI breakpoints (clarithromycin, amikacin, moxifloxacin, and linezolid), the main difference in the results of both studies lies in the resistance rates to moxifloxacin, which are significantly higher in our study (See Table 1: 45.5% and 38.2% vs. 17.6% and 11.3%). Studies from other regions, such as the one conducted by Siran Lin et al. in China, show different susceptibility profiles, highlighting the difference in the resistance rates of moxifloxacin and linezolid, which are lower in our study [15]. These findings may be significant, as although moxifloxacin and linezolid generally show limited activity against MAC, they can serve as important second-line treatment options. Therefore, they highlight the need for additional susceptibility studies in different regions due to the variability in resistance rates of these antibiotics.

The results for the other antibiotics with CLSI breakpoints, clarithromycin and amikacin, reinforce their role as first-line therapeutic options. Clarithromycin is the cornerstone of treatment for MAC infections, and amikacin is recommended as an addition to the standard three-drug regimen (macrolide, ethambutol, and rifabutin) in cases of severe or refractory lung disease, given its potential renal and auditory toxicity [10,11,16]. In our study, both antibiotics exhibited a susceptibility of over 90%, consistent with those previously mentioned [12,15] and studies conducted in other parts of the world, such as Germany [17], Korea [18], China [19], Sweden [20] and Israel [21], albeit with slight variations.

With regard to the genotypic detection of clarithromycin and amikacin resistance using the GenoType NTM-DR kit, the low number of resistant isolates limits our ability to properly analyze the data. However, it is important to highlight that it is a valuable tool for routine use due to the reduced response time compared to AST. In our study, the two clarithromycin-resistant isolates carried mutations in the rrl gene (23S rRNA). The use of the GenoType NTM-DR kit provides a rapid method for detecting these mutations, which could potentially allow for quicker adjustments to the treatment regimen, facilitating more tailored therapeutic strategies. Nevertheless, AST remains essential, as a negative result with the GenoType NTM-DR does not exclude the resistance to these antibiotics through other mechanisms, as was observed in our study with amikacin.

Macrolide resistance is generally low, and refractory MAC lung disease is commonly caused by the reinfection with new MAC strains rather than the emergence of resistance [22]. However, recent studies [23] report slightly higher resistance rates to clarithromycin [20%] compared to the previously mentioned studies. This underscores the importance of continued surveillance for the potential emergence of resistance in the future, including performing AST on clinically significant isolates and conducting similar studies in different regions worldwide.

Concerning the differences between species, our findings highlight significant differences in susceptibility profiles between *M. avium* and *M. intracellulare*, particularly for amikacin and linezolid, which showed markedly higher resistance in *M. avium*. These results are consistent with previous reports indicating species-specific variations in drug susceptibility within MAC [12,13]. The reasons for the different resistance patterns are not yet well established, although one hypothesis could be that genetic variations are influenced by environmental factors. While our study found *M. avium* to be more resistant, other studies conducted in geographically distinct regions, such as Korea, have reported *M. intracellulare* to be more resistant [13]. These geographical and environmental differences appear to play a significant role in shaping the susceptibility patterns between *M. avium* and *M. intracellulare*. Although neither amikacin nor linezolid are first-line options under normal circumstances (amikacin is used in severe or refractory cases, and linezolid is a second-line antibiotic), in specific situations, they may still be useful antibiotics. Therefore, the implications of these differences are clinically significant, as they suggest that species-level identification may be important for selecting the most appropriate treatment regimen for MAC infections.

With regard to the other antibiotics, the absence of breakpoints provided by CLSI prevents a comparative analysis of susceptibility; however, the distributions of MIC values can still be analyzed. Although this approach does not offer the same level of clinical certainty as when using breakpoints, it provides valuable information about the resistance patterns of the isolates to the antibiotics tested. However, it is important to note that the absence of breakpoints limits the ability to make precise treatment recommendations based on our MIC data. Further research is needed to develop or adapt appropriate breakpoints for MAC infections, which would help improve the interpretation of results and guide therapeutic decisions.

Rifampin and rifabutin are both commonly used as first-line treatments in combination with macrolides and ethambutol. However, rifabutin is preferred [24] due to its ability to reach higher tissue concentrations and demonstrate greater activity (albeit with more adverse effects) [25]. This is confirmed in our study, as the median MIC is significantly lower for rifabutin. Ethambutol is also used as a first-line antibiotic in treatment regimens. However, both in our study and in the previously mentioned literature [12,15,17,20], the MICs for most MAC strains are clearly above the established MIC cutoff for ethambutol-susceptible *M. tuberculosis* isolates. The use of ethambutol is primarily justified by its ability (through unknown mechanisms) to prevent the development of resistance to clarithromycin, a function that neither depends on nor correlates with its MIC [26]. Streptomycin is an alternative to amikacin in cases of severe lung disease [27]; however, the high MICs observed in our study suggest that it may not be the most appropriate therapeutic option. For both antibiotics, in our region, *M. avium* is significantly more resistant than *M. intracellulare*, which should be taken into consideration. The remaining drugs studied were isoniazid, SXT, ciprofloxacin, ethionamide, and doxycycline. All of them exhibited relatively high MICs, which is consistent with their not being used in the treatment of MAC. Although ciprofloxacin and ethionamide displayed significantly different MICs between *M. avium* and *M. intracellulare*, this variation holds no clinical relevance as these antibiotics are not recommended for MAC therapy.

A limitation of our study is the lack of clinical data correlating in vitro MIC results with treatment outcomes. While susceptibility testing provides valuable guidance, its predictive value is established only for a few drugs. Future studies should aim to bridge this gap by integrating microbiological and clinical data to refine treatment algorithms. Another limitation is the small number of clarithromycin and amikacin-resistant strains we obtained, which limits our ability to compare them with the genotypic results. Although valuable studies in this field exist, such as the one conducted by Hee Jae Huh et al. [28], it would be interesting to perform this comparison with isolates from our region. Furthermore, the single-center design may limit the generalizability of our findings to other regions, as resistance patterns and species distribution can vary geographically. Additionally, no corrections for multiple comparisons were applied in the statistical analysis, as this study was exploratory in nature. The reported *p*-values should therefore be interpreted with caution, and further studies are needed to confirm these findings. Lastly, although we included both pulmonary and extrapulmonary isolates, the focus on a limited number of extrapulmonary cases may introduce a bias that affects the representativeness of our data. This emphasizes the need for future studies with a more balanced inclusion of clinical samples to better understand resistance profiles across different MAC infection sites.

In conclusion, this study provides important insights into the antimicrobial susceptibility profiles of *Mycobacterium avium* complex (MAC) strains isolated in southern Spain and contributes to the expansion of comparable AST data in MAC. Our findings emphasize the regional variability in resistance patterns, particularly regarding moxifloxacin and linezolid, highlighting the need for localized susceptibility testing. The statistically significant differences in MIC distributions for amikacin, linezolid, ethionamide, ciprofloxacin, and streptomycin between *M. avium* and *M. intracellulare* underline the importance of species-level identification, highlighting the heterogeneity in resistance mechanisms among MAC species. Given the variability observed, both in our study and others, the development of localized treatment guidelines based on species-specific MIC data would be a valuable step forward in optimizing treatment regimens for MAC infections. Additionally, future research should aim to integrate clinical outcome data with susceptibility testing to better inform therapeutic decisions. However, it is important to note that our findings are preliminary and require further validation through larger, multicenter studies to confirm their applicability and impact on clinical practice.

## Figures and Tables

**Table 1:** Antimicrobial susceptibility of subspecies of MAC *.

Species	N	Interpretation	Clarithromycin [%]	Linezolid [%]	Moxifloxacin [%]	Amikacin [%]
*M. avium*	66	S	64 [96.9]	2 [3.0]	16 [24.2]	54 [81.8]
		I	0 [0.0]	18 [27.3]	20 [30.3]	6 [9.1]
		R	2 [3.1]	46 [69.7]	30 [45.5]	6 [9.1]
*M. intracellulare*	68	S	68 [100]	12 [17.6]	12 [17.7]	68 [100]
		I	0 [0.0]	36 [52.9]	30 [44.1]	0 [0.0]
		R	0 [0.0]	20 [29.4]	26 [38.2]	0 [0.0]
Total	134	S	132 [98.5]	14 [10.4]	28 [20.9]	122 [91.0]
		I	0 [0.0]	54 [40.3]	50 [37.3]	6 [4.5]
		R	2 [1.5]	66 [49.3]	56 [41.8]	6 [4.5]

S—susceptible; I—intermediate; R—resistant. * Breakpoints provided by the CLSI Guidelines [13].

**Table 2:** Median MIC values [µg/mL] and Wilcoxon rank-sum test *p*-values for antibiotics without CLSI breakpoints, stratified by species.

Antibiotic	*M. avium*	*M. intracellulare*	Total	*p*-Value
Rifabutin	0.25	0.50	0.5	0.23
Ethambutol	8	4	4	0.36
Isoniazid	4	4	4	0.21
Rifampicin	2	4	4	0.59
SXT	4/76	4/76	4/76	0.55
Ciprofloxacin	32	8	8	0.02
Streptomycin	32	16	32	<0.0001
Doxycycline	32	32	32	0.17
Ethionamide	2.5	7.5	5	0.015

## Data Availability

The raw data supporting the conclusions of this article will be made available by the authors upon request due to ethical reasons.
